# “They Were Saying That I Was a Typical Chinese Mum” : Chinese Parents’ Experiences of Parent-Teacher Partnerships for Their Autistic Children

**DOI:** 10.1007/s10803-022-05748-z

**Published:** 2022-09-23

**Authors:** Jodie Smith, Aspasia Stacey Rabba, Lin Cong, Poulomee Datta, Emma Dresens, Gabrielle Hall, Melanie Heyworth, Wenn Lawson, Patricia Lee, Rozanna Lilley, Najeeba Syeda, Emily Ma, Julia Wang, Rena Wang, Chong Tze Yeow, Elizabeth Pellicano

**Affiliations:** 1 Macquarie School of Education, Macquarie University, 2109 Sydney, NSW Australia; 2 Positive Partnerships, Chatswood, Australia; 3 School of Allied Health, Human Services and Sport, La Trobe University, Melbourne, Australia; 4 Department of Psychology, Counselling and Therapy, School of Psychology and Public Health, La Trobe University, Melbourne, Australia; 5 Department of Clinical, Educational and Health Psychology, University College London, London, UK; 6 School of Educational Psychology and Counselling, Faculty of Education, Monash University, Melbourne, Australia; 7 Reframing Autism, Sydney, Australia

**Keywords:** Cultural and Linguistic Diversity, Chinese Parents, Parent-Teacher Partnerships, Autistic Students, Participatory Research

## Abstract

Effective parent-teacher partnerships improve outcomes for autistic students. Yet, we know little about what effective partnerships look like for parents of autistic children from different backgrounds. We conducted interviews with 17 Chinese parents of autistic children attending Australian kindergartens/schools to understand their experiences. Parents appreciated the acceptance, opportunities and supports they received in Australia. They had high expectations of children; expectations not often shared by educators. Parents were respectful of teachers’ expertise and polite and undemanding in interactions. Nevertheless, parents were frustrated by inconsistent teaching quality and inadequate communication. Navigating systems was also challenging and parents faced discrimination from teachers and their community. Recommendations include fostering open home-school communication, proactively seeking parents’ expertise about children and explicitly scaffolding parents’ self-advocacy.

Effective partnerships between teachers and caregivers – namely those with open communication, trust, advocacy and respect (Turnbull et al., 2015) – can substantially improve children and young people’s success in and out of school (Sheridan et al., 2012). As autistic students may benefit from consistent approaches across home and educational settings (Azad & Mandell, 2016; Simonoff et al., 2012), such partnerships may be especially important for them (Lilley, 2019). Unfortunately, parents of autistic children often report substantial challenges with their children’s education, highlighting a lack of access to autism-specific knowledge, expertise and support for their children, poor communication channels, adversarial relationships with teachers and ineffective collaboration with teachers and education settings (Lilley, 2019; McNerney et al., 2015). Furthermore, despite feeling they know their children best, parents often describe feeling not listened to and excluded from classrooms, resulting in them feeling isolated and unsupported (Lilley, 2014; Makin et al., 2017). It is likely that additional challenges exist which further impact family engagement in parent-teacher partnerships for Culturally and Linguistically Diverse (CALD) parents of autistic children but we have limited data, especially in an Australian context.

## Australian Demographics

Australia is a broadly Westernised but highly multicultural country, with 30.0% of the population (7.6 million residents) born overseas (Australia Bureau of Statistics, 2021). Behind the United Kingdom and India, China now provides the third highest number of overseas-born residents to Australia, comprising 2.5% of the 2020 population, with twice the number of Chinese-born people living in Australia in June 2020 (650,640) compared to a decade earlier (Australia Bureau of Statistics, 2021). International migration – when one moves from one country to another (Sinha, 2005) – is itself a challenging experience and, for various reasons, migrants who have an autistic child face increased stressors (Kim et al., 2020; Lim et al., 2020).

As Australia is home to a large proportion of migrants from Chinese backgrounds, we need to understand how best to assist these families, especially as they may be uniquely different to other migrant groups. For example, China does not permit dual nationality so these migrants can be less willing to forgo Chinese citizenship and risk future exclusion from their home country (Stevens, 2018). Indeed, recent data highlights low rates of naturalisation for Chinese migrants living in Australia when compared to other overseas-born groups (Pan, 2020). Consequently, Chinese migrants may be more likely to experience ongoing disruptions to their family and work lives during their extended periods in limbo between countries (Stevens, 2018). Moreover, trying to maintain relevancy and reduce marginalisation in both countries concurrently may also pose a challenge (Gao, 2006). When families have an autistic child, this temporariness may be even more impactful in relation to accessibility and consistency of funding and/or familial supports. Since education is a universal and extended experience for all parents and children, understanding what might improve parent-teacher partnerships is an important first step in supporting migrant families of autistic children. So, here we focus on experiences of parent-teacher partnerships for migrant families of autistic children from Chinese backgrounds living in Australia.

## Culture, Caregiving, Education and Autism

Exploring culture in the context of caregiving, autism and parent-teacher partnerships is also important since culture influences people’s views and experiences in each of these spheres. Migrant Chinese parents have been found to be respectful of teachers and observant of distinct role boundaries (Collignon et al., 2001; Denessen et al., 2007; Lai & Ishyama, 2004). These parents have likewise reported feeling uneasy dealing with teachers (Lai & Ishyama, 2004) and reticent to voice concerns when dissatisfied with services for their children with disabilities (Liu & Fisher, 2017). Additionally, migrant parents are often faced with language barriers, fewer social supports and unfamiliarity with education systems and teaching approaches (Haines et al., 2018; Lai & Ishyama, 2004; Wang & Casillas, 2012).

Cultural views of caregiving and autism similarly add complexity. Whilst parents are highly respectful of teachers’ roles, they still feel acutely responsible for ensuring their children’s progress, especially academically (Li & Yeung, 2017; Shorey et al., 2020; Wang & Casillas, 2012). Frustration with inconsistencies in teachers’ experience and skills and low/inappropriate expectations of autistic students has previously been reported by migrant Chinese parents of children with disabilities (Lai & Ishyama, 2004). Chinese parents also face autism stigma (Kim et al., 2020; Tang & Bie, 2016), with the concept of ‘losing face’ (i.e., the reduction of interlinked familial and individual dignity and status) playing a prominent role in stigmatisation in native and migrant Chinese populations (Huang & Zhou, 2016; Liao et al., 2019). Since Australian teachers are largely white, female and monolingual (Australian Institute for Teaching and School Leadership, 2020; Evans, 2011), migrant parents also face potential discriminatory treatment from teachers whose views about parent involvement and education are likely to reflect their own Western-centric experiences and training (Bakker et al., 2007).

All of the aforementioned factors are likely to contribute to what effective parent-teacher partnerships looks like for Chinese parents, as well as how these partnerships develop. Despite theoretical knowledge of the difficulties Chinese parents of autistic children can face when interacting with teachers and schools, there is virtually no research examining their experiences in Australia. Recent research has explicitly articulated how positive relationships between teachers and Chinese parents can be a source of parental support as well as ensuring autistic children’s success in schools (Zhao & Fu, 2022), so finding out how to foster positive parent-teacher relationships is important for several reasons. In this study we sought to elicit the first-hand accounts of Chinese parents living in Australia as they navigated schooling for their autistic children.

## Method

### Community Involvement

Participatory or co-produced research supports collaboration across researchers, practitioners and community members (Hickey et al., 2018). This type of research aims to ensure studies are respectful, ethical and responsive to the needs, preferences and principles of the communities at the centre of the research (Collins et al., 2018). This study adopted a participatory approach, which operated at several levels. To begin, autistic scholars and advocates who were themselves also autistic parents of autistic children (GH, MH, WL) worked collaboratively with non-autistic researchers (JS, SR, PD, RL and EP) to secure the research funding for this project and design the initial study. Next, the team – and JS in particular – worked together with ED - to assemble a Chinese-specific parent Advisory Group (AG), consisting of five Chinese parents of autistic children (4 mothers, 1 father, all co-authors on the study), researchers and professionals, including a Mandarin and Cantonese speaking interpreter. This AG met four times over the duration of the project, overseeing the recruitment of participants, as well as study design, implementation and dissemination. Their involvement safeguarded that the project was sensitive to, and relevant for, the Chinese autism community (i.e., ensuring the suitability of questions asked of parents and the appropriate messaging and disseminating of research findings). Chinese AG members were paid for their time and expertise.

### Recruitment and Participants

Participating parents had to be ≥ 18 years and self-describe as being from a Chinese background. There were no English-speaking requirements. All children of participating parents had received an independent clinical diagnosis of autism and were engaged in education (early education, primary or high school, or home-schooling). Participants were recruited through formal and informal networks (i.e., word of mouth, Chinese community groups etc.). All recruitment and interview materials (i.e., demographics questions, parent interviews and recruitment flyers) were available in English, Simplified Chinese and Traditional Chinese. Eighteen parents were recruited and interviewed (including one mother-father couple) with one interview omitted from analysis as it was subsequently revealed that the autistic student had recently completed school.

Of the 17 parents, most were female (*n* = 14; 82.4%) and had post-school qualifications. Almost half (*n* = 7) of parents were sole parenting. Most parents were born in China (*n* = 12; 70.6%), with the remaining born in Hong Kong (*n* = 4; 23.5%) and Malaysia (*n* = 1; 5.9%). Together, parents had 19 autistic children (*n* = 15 males, *n* = 4 females). At the time of interview, autistic children were on average eight years of age (range 2–17, SD = 3.91). Children largely attended mainstream kindergarten/school settings (*n* = 14) with two children in special schools and the remaining three children in other/dual settings, (i.e., where children split their weeks between different types of educational setting). See Table 1 for parent, child and family characteristics.

**Table 1:** Characteristics of Chinese Families involved in the Study (*n* = 17^1^ parents, n = 19 children)

	N (%)/M (range, SD)
**Child characteristics**	
Gender	
Female	4 (21.1)
Male	15 (78.9)
Age at Parent Interview	8 (2–17, 3.91)
Average Age of Diagnosis^2^	4 (1–10, 2.80)
Education Setting	
Kindergarten/Preschool	4 (21.1)
Mainstream	10 (52.6)
Disability Specific	2 (10.5)
Other^2^	2 (10.5)
Missing	1 (5.3)
School Year	
Primary	8 (42.1)
Secondary	3 (15.8)
Missing	4 (21.1)
NDIS plan in place	
Yes	18 (94.7)
**Parent characteristics**	
Gender	
Female	14 (82.4)
Male	3 (17.7)
Age at Parent Interview	43 (31–56, 6.95)
Education Level	
Post-school (i.e., diploma/certificate)	1 (5.9)
College	1 (5.9)
University Degree	13 (76.5)
Post-graduate	2 (11.8)
Employment Status	
Full-time	2 (11.8)
Part-time	4 (23.5)
Homemaker/Full-time Parent	4 (23.5)
Self-employed	2 (11.8)
Unable to work due to disability	1 (5.9)
Missing	4 (23.5)
Country of Birth	
China	12 (70.6)
Hong Kong	4 (23.5)
Malaysia	1 (5.9)
Identified Culture	
Chinese	10 (62.5)
Asian	2 (11.8)
Hong Kongese	1 (5.9)
Chinese/English	1 (5.9)
Australian Chinese	1 (5.9)
Missing	1 (5.9)
Year moved to Australia	
1980–1989	2 (11.8)
1990–1999	1 (5.9)
2000–2009	5 (29.4)
2010-	8 (47.1)
Missing	1 (5.9)
**Family characteristics** ^3^	
Number of Autistic Children	
1	13 (81.3)
2	3 (18.8)
Parenting	
Single parent	7 (43.8)
Two parents	5 (31.3)
Missing	4 25.0)
Extended Family living in Australia^4^	
Yes	4 (25.0)
No	9 (56.3)
Missing	3 (18.8)
Household Income	
No current income	1 (6.3)
$1 to $25,000 per year ($1-381 per week)	2 (12.5)
$25,001 to $50,000 per year ($482–962 per week)	4 (25.0)
$50,001 to $78,000 per year ($963-1,500 per week)	1 (6.3)
$78,001 to $104,000 per year ($1,501-2,000 per week)	0 (0.0)
$104,001 or more per year (more than $2,001 per week)	2 (12.5)
Missing/ I’d prefer not to give this information	6 (37.5)
Languages Spoken at Home	
Cantonese	5 (31.3)
Mandarin	3 (18.8)
Chinese	1 (6.3)
Chinese languages and English	5 (31.3)
Shanghaiese	1 (6.3)
English	1 (6.3)

*Note*. NDIS = National Disability Insurance Scheme. ^1^one mother-father couple interviewed together; ^2^included dual schooling (i.e., attending both mainstream and special schools and support classes in mainstream); ^3^only provided for 16 households; ^4^examples included child’s grandparents, parents’ siblings/partners

### Procedure

This study was conducted from January to December 2021, during the second wave of the COVID-19 pandemic in Australia. Once parents had provided, informed consent, they each completed a background questionnaire (either online, over the phone or at the beginning of the interview). Each parent then took part in an in-depth Zoom interview, as pandemic-related restrictions at the time precluded the possibility of face-to-face interviews. Parents were asked about their experience of their child’s kindergartens/schools, interactions/involvement with teachers and ideal parent-teacher partnerships. Parents were also asked how they felt the Chinese community understood autism (see Supplementary Table 1 for full interview schedule). Parents were interviewed in their preferred language (Mandarin, *n* = 7; Cantonese, *n* = 6; English, *n* = 4) by someone from their cultural background [PL]. A separate interpreter was hired to translate Mandarin/Cantonese interviews. Interview recordings were transcribed verbatim using a transcription service. Parents were reimbursed for participating.

### Data Analysis

We followed Braun and Clarke’s (2006) method for reflexive thematic analysis within an essentialist framework in which our goal was to report the meanings and experienced reality of the participants. Once all interviews had been transcribed, one senior researcher [JS] immersed themselves in the data, taking notes on striking and recurring observations and applying codes to each transcript (managed in NVivo, version 12). To begin, JS developed and applied codes in discussion with EP. Next, JS generated a draft thematic map showing potential themes and subthemes and this map along with all relevant quotes was revised during multiple discussions with EP. Finally, the revised thematic map was reviewed by the broader team [SR, PD, GH, MH, WL, RL and NS] as well as members of the Chinese AG [ED, LC, ED, PL, EM, RW, JY and CY] prior to being finalised. Analysis was therefore iterative and reflexive in nature (Braun & Clarke, 2006, 2019).

**Fig. 1:**
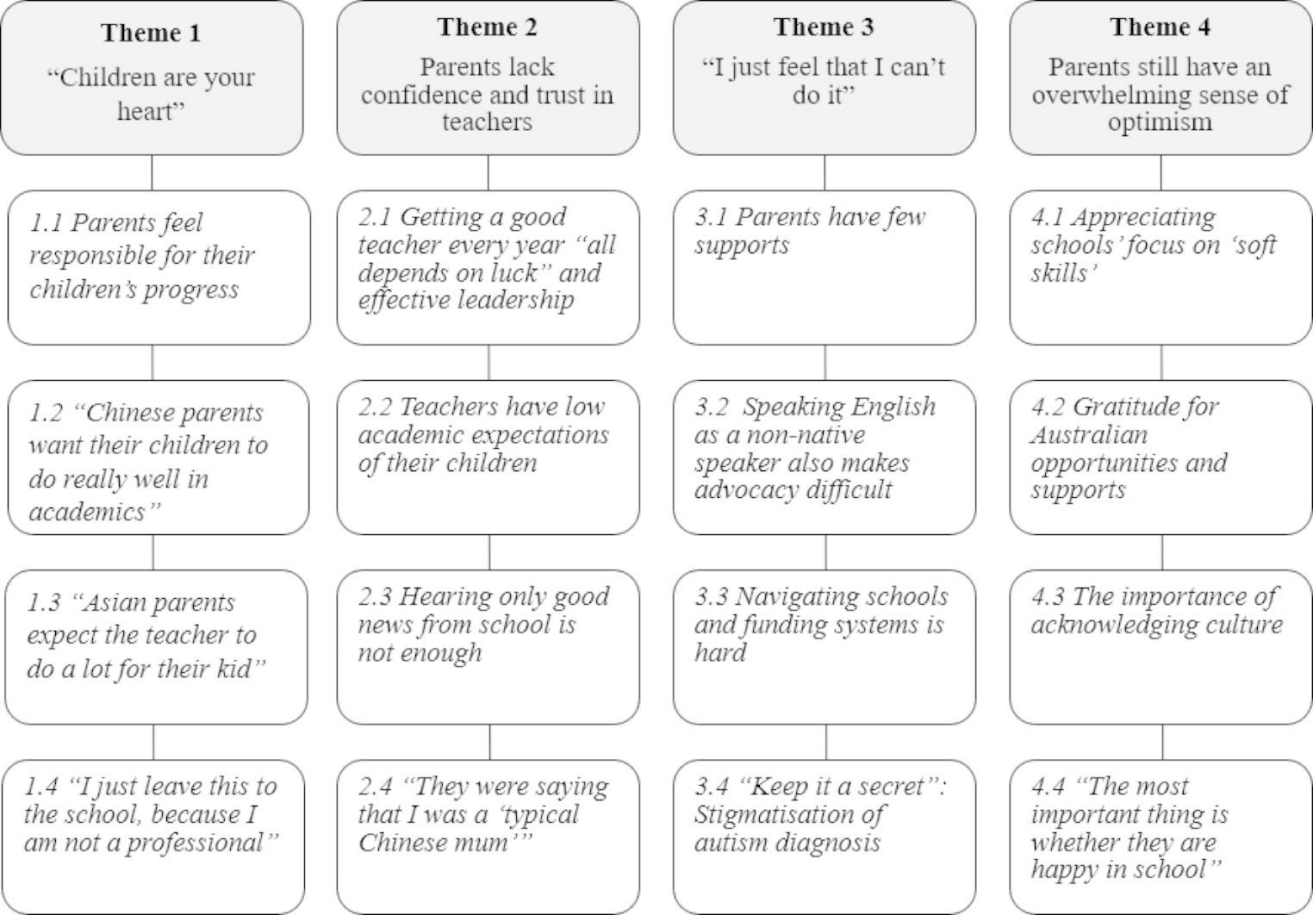
Chinese parents’ experiences of educating their autistic children: themes and sub-themes

## Results

Key themes and associated subthemes are presented in Fig. 1. Illustrative quotes (with participant random IDs i.e., [17]) are provided below to show the breadth of responses.

### Theme 1: “Children are Your Heart”

Parents were so grateful that Australian teachers were “very patient with the children and respect the children” [59] and that they were “very kind and helpful” [46]. Overall, parents just wanted “the best” for their children [42]. As one parent put it: “Every family has only one or two children and those children are your heart” [39]. As part of this child-centric view, parents felt *autistic children’s progress was the “responsibility of the parents”* [10] (subtheme 1.1). So, if children were not progressing then it was up to parents “to actually pay more attention to their child”, “to observe the child and find the problem” [64]. To support their children effectively, parents felt that they needed to take “initiative to do things… to rely on [themselves]… and do self-learning” [70]. This sense of parental accountability was reportedly common in China: “Chinese teachers… they won’t attribute anything to the children themselves. They would think that it’s the parents’ responsibility” [73]. Parents’ devotion to their children and their child’s learning, meant that they “spent a lot of time” [67] “just focused on [their] child” [59]. Although parents were “happy to invest a lot of time” [64] in their children, it required parents to “work very hard” [48]. As one parent emphasised: “I wouldn’t discount as parents how hard we have tried” [39].

Parents went on to explain that *“Chinese parents, they still want their children to do really well in academics”* [39] (subtheme 1.2). High academic expectations were explicitly linked by our respondents to Chinese cultural values. Parents often mentioned how competitive schooling was in their home countries: “In China, the competition is very fierce” [27]; “In Hong Kong, there is a lot of competition” [46]. Parents therefore felt it was important to support their children to achieve their potential: “As parents, we know his abilities, so we try to push hard on every single task” [39]. Even for parents who were not as focused on academic achievement per se, accomplishment was still prized: “I am not a high-pressure parent, but we still have expectations” [23]. They reported how school learning was regularly supplemented through learning at home: “Asians go to tuition, coaching” [34]. One parent explained that he “sent [his son] to coaching” because Australia was “academically, too tolerant, too relaxed” [48]. Parents often helped their children “with study or academic performance” [46] at home.

Consonant with high expectations for their children, *“Asian parents expect the teacher to do a lot for their kid”* [10] (subtheme 1.3). Parents wanted their children to have a “high standard education”, to attend “a very high-quality school” [39]. If parents felt their child’s educational setting was not suitable, they would readily move children: “We changed kindergartens five times; he went to different kindergartens because we were not satisfied” [65]. Parents expected that educators “teach [their children] properly” [10] and engage with their children, “asking [them] in depth as to how [their] day is” [34]. Parents felt that teachers should be “more responsible and watch more closely” [48], providing them with “constant updates” and “more feedback” [20]. One parent had especially high expectations of schooling in Australia: there are “20 to 30 kids” in classes in Australia compared to “50 to 60 kids in one class” in China, so Australian teachers “should have more time… double the time” [48].

Despite expecting much of Australian teachers, parents expressed disappointed in their work ethic when compared to teachers in China/Hong Kong. One parent explained: “In Australia, the working culture is you just finish your job, you go home when your shift ends. But in China, you stay in your office until you finish” [64]. Another parent also felt that Australian teachers think: “‘Oh, time to go’ but in Hong Kong they will try to stay back to finish” [10]. Parents felt that teachers could and should work harder for their children: “I can’t say they’re irresponsible, but I feel like they can do more” [48]. One parent reflected: “Maybe if I were in China, I would have expected more” [67]. This sentiment was not just about putting in the hours but extended to how much they felt they cared about their children: “They might regard teaching just as a job… they normally just follow the standard procedures and don’t spend more energy or passion in finding out special needs from the kids with autism” [73].

Even though parents were the experts on their children, they maintained distinct boundaries between home and school: *“I just leave this to the school, because I am not a professional”* [46] (subtheme 1.4). This delineation was reportedly common in China: “You don’t need to take the initiative; the teacher would take the initiative and talk to you” [59]. Parents firmly believed teachers were the “professionals” [39] with “particular qualifications to be able to work in the sector” [20] – and thus expertise and experience that was distinct to their own. As one parent remarked: “Parents are just ordinary people, so they rely on the school to teach the parents how they educate their kids” [48]. Accordingly, they felt that they had no “authority on what to teach at school” [73] as schools “follow guidelines” [42] and “have the school curriculum” [64]. This was despite parents being quietly aware that they had expertise and experience that might be beneficial to share with teachers: “I knew very clearly what my child could do. If you ask me for experiences, I mean, I can give you a lot of examples” [64].

Parents were also profoundly “respectful” [39] of parent and teacher roles. They were acutely aware of not wanting to be perceived as “overbearing” [14] in their interactions with teachers. They often reported feeling “too shy to ask” [48] for things, or “ask for too much” [59] and reported “always apologising” [23] and being careful “not to interrupt” [39], and not “arguing with the teacher” [14]. Nor did they want “to be impolite” [42]. When they were unsure of the “expectations”, they worried about “doing the wrong thing” [34]. Parents clearly did not want to impose on teachers’ time or be a burden. One parent described how he was expecting an invitation to the school but, when none arrived, he decided not to follow up as he “did not want to push and add to [their] load” [48]. Even when parents did speak out, they made sure they only “push(ed) in a respectful way” [39].

### Theme 2: Parents Lack Confidence and Trust in Teachers

Despite their respect for clearly defined roles, parents often reported frustration that *getting a good teacher every year “all depends on luck”* [42] *and effective leadership* (subtheme 2.1). The idea of good fortune in relation to good teachers was common: “I was lucky. I got one teacher… she’s got 20-years’ experience” [10]; “If you have a good teacher then you are lucky, and if you have a bad teacher, you are unlucky” [42]. Parents valued effective leadership but, again, this was perceived to be largely a matter of chance: “I spoke to the headmaster; I spoke to the person who was responsible for allocating teaching aids and I was lucky” [59]. Although variability across teachers’ experience and skills is to be expected, parents found it frustrating that schools appeared aware of differing teacher quality: “I talked to the vice-principal, and the principal told me that, don’t worry about it because next year he will give me a better teacher” [42]. Parents perceived teacher quality as having a knock-on impact on children’s experience and desire to engage in education: “For the mainstream school, I found that she’s reluctant. She’s finding excuses of not going to this school” [27]. Another parent said, “He loved going to (previous) school but… I feel like he is less keen. If he has a choice, he would choose not to go to school” [48].

Parents were disappointed by what they perceived to be *teachers’ low academic expectations of their children* (subtheme 2.2). This was especially in the context of disability-specific schools with one parent stating that her son had not “done much academics in special school because they’re only doing the behaviour correction stuff” [39]. Another parent reported being told by the teacher that “you cannot ask autistic children to do much” [59]. One parent simply said: “The teacher gave up on my son” [70]. Parents also spoke of how their children were “bored… not engaged” [39] due to not being challenged academically at school. Parents reflected on whether expectations of their children would be higher if they were still in China:

If [child] were in China, I would have expected him to go to university. Although my child has some problems in reading and comprehension… in China, they could actually give him a lot of pressure and then he could study harder and then eventually he may have the chance of going to university [59].

Parents also complained that all they *“hear from school is the good news*” [39] (subtheme 2.3). While they appreciated “positive feedback”, they expressed the view that “Australian teachers don’t want to talk about the bad things” [42]. They contrasted this experience with their experience of schooling in China where teachers would “tell you your kids are doing good in school, but they then would also tell you your kids may not be doing quite well” [27]. Parents even said “it is quite common” [65] for caregivers in China to stay with their children at school all day. Yet, parents felt discouraged from attending Australian schools: “They [teachers] just tell you that the classroom is not for you; it’s for the students and the teacher” [70]. One parent recounted being told “okay, go home, mum” [23].

With teachers in Australia perceived to be avoiding telling them “any negative things” [14], parents felt that they were not getting a “a true reflection of how [their] kids are doing in school” [27]. Parents also described “inconsistency” across teacher feedback. One parent described how the “generic report is generally very positive” but “individualised feedback” had made it clear that their child “didn’t do as well as [they] had thought” [70]. Another reported explicitly requesting that the teacher “give [her] some negative things” about her child [59]. The incomplete picture parents felt that they had of their child’s education was compounded by having “zero idea” [34] about what their child learns at school and no “clue how to find out” [70]. This experience was described as distinctly different to Chinese education, which was described as providing many more opportunities to learn first-hand information about their children – either through direct communication between parents and teachers (“we have this WeChat group… so the teachers can post updates any time, and the parents can discuss issues more frequently” [20]) or through “technical things like apps” [64] or “live cameras so that the parents can monitor what’s going on in the classroom” [20].

It was not just the perceived absence of interaction between parents and teachers that was challenging; parents also found that the parent-teacher “communication channel was very frustrating” [34] in general. When some parents “asked for [school] contact details” they were told that they “need to speak to the office” [27]. Parents stressed how important their own “privacy” [65] and their “child’s privacy” [59] was, so using generic communication channels was problematic for several parents. One parent reflected: “You can’t e-mail the teacher, you have to send an e-mail to the reception” so “with the psychologist report and everything, I have to send it to the reception. And it’s exposed to everyone. Oh my God, I want to keep that private” [34]. Since parents felt they were neither given open and honest feedback about their children, nor encouraged to attend school in person, one alternative way for them to gather information was to “volunteer in the school” [59] where: “I can observe and find out how she’s doing in school” [46]. As one parent described: “I actually took a very active role in taking part in the school’s parent helper programme… I have the chance to see how my kid is going with the school’s life” [73].

There were several reports of parents experiencing stigma which shook their confidence in teachers even further. When one parent tried to advocate for her son during a school enrolment interview, she described being racially stereotyped by the school coordinator: “So, at the beginning, when I dealt with him, *they were saying that I was a ‘typical Chinese mum’* (subtheme 2.5) … what he said and what he did was kind of making me upset” [42]. Another mother reported being judged about her single parent status:

They told me that they were happy to apply for some benefits, but then they told me that because I was a single parent, even if you took these benefits, the child would not have a father to enjoy. This made me quite angry… I don’t know why they discriminate against us [67].

### Theme 3: “I Just Feel That I Can’t Do It” [27].

Advocacy was especially difficult for these parents since they *had few supports* (subtheme 3.1). Many parents worked “full-time” [10] and “long hours, over ten hours of work each day” [48], often with “both the mum and dad needing to work” [64]. This meant parents frequently struggled to attend schools, especially during working hours. One parent said: “I need to work; I don’t have much time” [67]. Another agreed: “I’m working full-time, I can’t contribute much” [34]. While some parents were helped by family members living in Australia (“We have work, during weekdays, they [grandparents] will come to help us” [73]), others were not: “Normally if you have a child, you have your old parents, like a grandma or grandpa, who could actually look after your child, or support you” [65]. Many parents (mainly mothers but some fathers) were also solo parenting in Australia so were especially impacted by a lack of extended family support. The pandemic decreased access to supports even further as people (including family members) could not easily enter Australia: “We came to Australia about two years ago. My husband is still in Hong Kong so it’s three of us here” [70].

Parents’ lack of familial support was further compounded when other supports within schools were not approved: “The psychologist cannot come to observe in the classroom. That’s not allowed” [34]. As with inconsistencies in the quality of teaching staff, whether professionals were allowed into schools varied across schools – “Some schools welcome visitors especially from a speech pathologist… [my] school didn’t want this to happen” [70]) – and different contexts within the same school – “For other meetings, I always brought with me the psychologist, and then for this meeting I couldn’t bring with me the psychologist” [42].

*Speaking English as a non-native speaker also made advocacy difficult* (subtheme 3.2): One parent stated: “When I go to the school and pick up the child, I seldom talk to the teacher, because my English is limited” [65]. Another parent mentioned that when parents “form a group… it’s easier to advocate for your child” but forming a parent group was difficult for them because their “[English] ability is limited” [46]. Parents were sometimes offered interpreters: “For the meeting with the teacher, they ask whether you’ll need an interpreter or not” [67]. Some found these interpreters valuable – “They arranged an interpreter, and it was quite good” [65]) – while others felt meetings were too short - “maybe it is only 15 or ten minutes” [27] – to use interpreters in a meaningful way, especially because having an interpreter “doubles the time” [48].

*Navigating school and funding systems was hard for parents* (subtheme 3.3), with parents’ limited knowledge about systems, as well as limited understanding about autism (both pre- and post-diagnosis) contributing to these difficulties. Limited understanding of the Australian education system, including knowing their own and their child’s rights, meant parents could not be proactive: “Sometimes I don’t know what to advocate for and we don’t know the pathway” [23]. One parent mentioned that she did not know how to “enrol [her child] in a (mainstream government) school” so by the time she did enrol, the school “was full” [10]. Another parent had her child’s mainstream government school enrolment rejected… “so he stayed at home for the whole entire month” since she “didn’t know [she] had a right to ask for public school” [39].

Regarding autism knowledge, one parent stated that, in Chinese, “autism is a literal meaning of the phrase itself. It means isolated from the external world and not communicating with other people” [73] – so that is how community members perceived autism. Parents also described different beliefs that they, their family or their community held about the causes of autism. Some felt that “autism is kind of like a genetic problem, and then if your child has autism, maybe one of your parents has some extent of autism as well” [42], while others felt that it was due to some post-natal injury – “My husband believes that because they used forceps (in labour), it might have caused brain damage to my child, that’s why my son has this autism” [70] – or environmental factor(s) – “I think autism is caused by different causes – 60–70% from environmental causes and the rest, 30%, is natural causes” [64]. They also reported an apparently widely-held belief that autism “will get better naturally by itself” [46], that it is something the child will “outgrow” [34] and will “go away when [the] child grows up” [67].

*Stigmatisation of autism diagnosis also meant parents “keep it a secret” [70]* (subtheme 4.5). Parents reported wanting to “save face” [46] and were “afraid to see [their] friends (in China) because [they] don’t want to get embarrassed” [59]. This shame was sometimes reinforced by the feeling of being ostracised by others in their community because of their child’s diagnosis: “I can feel parents, once they know my son’s condition, they try to stay away from us, which is very heart-breaking” [39]. In response to being asked what specific factors might influence a Chinese parent not seeking or accepting a diagnosis, one parent simply answered “pride” [34].

### Theme 4: Parents Overwhelming Sense of Optimism

Despite all these challenges, there was a strong sense of optimism in parents’ responses. They believed that Australian schools were not academically challenging enough for their children, but were nevertheless *grateful that schools focussed on ‘soft skills’* (subtheme 4.1) – on “kids’ personal ability, personal development, and they encourage them to cook, to live independently… for kids with disability, it’s very important for them to grasp these practical skills” [23]. Another parent echoed how Australian teaching was well rounded: “They try to develop all abilities for the children… what they teach children, the value, the ethics, is way more than academics can measure, which is better” [39]. Moreover, some parents felt that non-academic skills should be more of a focus as children move through school: “For my child, he needs to develop academically but then he’s now a teenager and he has different psychological or mental needs… they need to provide more support in this respect, especially for the autistic children” [59].

Similarly, parents reported being truly *grateful for the supports and opportunities afforded by Australia* (subtheme 4.2). They were thankful for the financial support provided that they felt would otherwise not have been available to them: “If I were in China, I would have gone bankrupt because I just couldn’t afford to look after a child like this. I’m so lucky that I’m in Australia” [67]. Parents were also sympathetic to the fact that “teaching is very stressful” [23] so were thankful for teachers’ compassion: “We want to show our appreciation for how good they have treated my child” [39]. Parents also felt that there were more options for their children in Australia, as they moved into adulthood – “In Hong Kong I just cannot see our future, I cannot see my child’s future, but here, in Australia, I hope that my child can find a job and I can see her future” [46]. They also spoke about a greater acceptance of difference: “The Australian community is more tolerant and welcoming to diversity” [39].

Although parents often reported feeling responsible for their own integration into Australian society – “I am actually an immigrant to Australia, so my feeling is that I should get assimilated to the Australian culture” [42] – they still felt it was *important for schools to acknowledge their culture* (subtheme 4.3). Parents appreciated when their culture was recognised in schools. One parent relayed how her school had asked whether it would be helpful if someone at the school could “learn some Chinese and talk to [her son]”, which she felt was a “great approach” [39]. Another articulated the benefits of cultural appreciation, observing that when teachers “can understand the Chinese culture, and the child’s background, they might be in a better position to provide care or support” [65]. Although not expected, parents still valued “translated e-mails or materials” [67] and “Chinese speaking teachers” [46]. They were keen for more to be done to promote this sense of cultural safety including celebrating “cultural festivals, like the Chinese New Year or the Monkey [King] Festival… if they could arrange such activities, of course I would be very interested” [65].

Optimism also related to their children’s happiness because, above all *“for autistic children, the most important thing is whether they are happy in school”* [42] (subtheme 4.4). Another parent said: “I want my children to get a high score, Because we are from a Chinese culture. But I don’t want them to purely focus on academics” [39]. Parents reported being content “as long as the child’s happy in the school, as long as [they] can learn something” [64]. Hence, when able, parents chose schools that they believed best suited their children, regardless of whether they were disability-specific or mainstream settings. One parent reflected: “I can see [her daughter] is happy to go there because in the special school, they focus on developing their skills and then they provide a lot of programmes catered to their needs. They don’t need to actually focus on their study or academic performance” [27].

## Discussion

This research provides first-hand accounts of experiences of education for Chinese parents of autistic children educated in Australia. Our parents were devoted to their children and felt responsible for their progress and happiness. They had high expectations of children, especially academically, but they felt these expectations were not often shared by educators. Parents were profoundly respectful of parent/teacher roles and described themselves as polite and undemanding in interactions. Parents were frustrated by inconsistent teaching quality and inadequate communication from schools. They also often faced stigma and discrimination from both teachers and the Chinese community, and had few resources to rely upon. Nonetheless, parents were extremely grateful for the supports and opportunities afforded by Australia and valued that their children received a holistic education. And, whilst they did not expect schools to provide culturally-specific resources, they spoke about the benefits of acknowledging their culture in schools.

### Conflicted Feelings Towards Education

Parents were ambivalent towards Australian teachers and schools. On the one hand, they were grateful that Australian teachers accepted their children and were caring and respectful towards them. They also valued that schools tried to equip their children with life skills – a sentiment shared by parents of autistic children in the UK (Makin et al., 2017; McNerney et al., 2015). Echoing earlier research with migrant Chinese parents of children both with (Lai & Ishyama, 2004; Liu & Fisher, 2017) and without disabilities (Collignon et al., 2001; Denessen et al., 2007), they were deferential towards teachers, respectful of role boundaries and reluctant to voice concerns too. Jegathessan (2009) similarly found that migrant Asian parents in the United States (US) were grateful for opportunities afforded by their adoptive country and did not want to be perceived as asking for more.

On the other hand, parents were disappointed by teachers. They experienced discrimination from teachers and their efforts to enrol their autistic children in schools were often met with resistance, aligning with reports from Somali-Canadian parents (Kediye et al., 2009). Parents were also frustrated by inconsistencies in teachers’ experience and skills and by their low/inappropriate expectations of autistic students. These issues have previously been voiced by myriad groups of parents of autistic children, including migrant Chinese parents living in Canada (Lai & Ishyama, 2004), British (McNerney et al., 2015) and Australian parents (Hodges et al., 2020; Lilley, 2013, 2014). What has not been so clearly articulated before is how parents felt that their children’s desire to engage in education was directly impacted (positively or negatively) by variable teacher quality, in relation to teacher’s knowledge, skills and experience of autism and dedication to supporting autistic children.

‘Child-centredness’ is commonly reported by Asian parents of autistic children (Shorey et al., 2020). We found Chinese parents felt accountable for their children’s educational progress and had high academic expectations for their children (Li & Yeung, 2017; Shorey et al., 2020; Wang & Casillas, 2012). Where parents still living in China reported taking on the responsibility of educating their autistic children because of the limited availability of supports (Liu & To, 2021; Zhao & Fu, 2022), it was the perceived low academic expectations within schools that led many parents in our study to provide more home-based educational activities (i.e., tuition, coaching and homework). Perhaps provision of more educational activities within the home was also driven by parents’ unfamiliarity with education systems.

Recent reviews in this field have similarly found difficulty accessing/navigating services key challenges for migrant parents of autistic children (Kim et al., 2020; Lim et al., 2020; Papoudi et al., 2020). Past research with non-migrant parents of autistic children has also reported how these parents struggled to navigate school systems too (Lilley, 2013; McNerney et al., 2015). What was noteworthy in this study was that parents’ unfamiliarity with school systems directly impacted children’s lawful right to access and participate in education – including the right to attend school full-time and receive reasonable adjustments (See Disability Standards for Education; Australian Government, 2005). A key difference for individual families therefore is the availability (or lack thereof) of different sources of information they can rely on for support when they struggle to navigate their autistic child’s education, and the corollaries of that access to, or absence of, knowledge.

Parents’ unfamiliarity and limited knowledge was compounded by inadequate, opaque communication from schools and limited opportunities to observe their children first-hand. Inadequate communication from schools is another common frustration shared by many parents of autistic children (Azad et al., 2018; Galpin et al., 2018; Lilley, 2019; Makin et al., 2017). Our Chinese parents both wanted and expected more frequent parent-teacher interactions through which they could gain important, timely information about their children. As with past research with non-CALD parents of autistic children, parents wanted to celebrate their children’s successes but also be informed about challenges (Stoner et al., 2005). Yet where non-CALD parents can feel schools focus too much on the negative aspects of their autistic children (Azad et al., 2018; Lilley, 2019), interestingly, Chinese parents felt the opposite to be true here. Effective parent-teacher communication is therefore not a one-size fits all.

Unfortunately, inadequate communication from schools, coupled with experiences of discrimination and inconsistent teaching quality, eroded parents’ trust and confidence in schools. Asian migrants of non-autistic children living in New Zealand have similarly indicated a lack of confidence in teachers for various reasons, including mistrust of teachers and communication barriers (Guo, 2005). To safeguard that their children were managing at school, parents gathered information themselves (i.e., through volunteering in the school). This increased watchfulness has similarly been reported by non-CALD parents of autistic children when trust in professionals has been lost (Stoner et al., 2005). Yet our parents had few other resources and supports (i.e., extended family/partners, available time during school hours etc.) so may be more likely to experience increased psychological impacts over time related to the burden of balancing childcare responsibilities and paid work with little familial/social support (Zhao & Fu, 2022).

### How Can We Develop Effective Parent-Teacher Partnerships for Chinese Parents?

As with past models of parent-teacher partnerships for non-autistic children (Keyes, 2000; Turnbull et al., 2015), successful partnerships for Chinese parents included good communication between parties (especially updates about children’s progress), professionals having appropriate knowledge and skills, as well as mutual respect and consideration of each other’s culture and values. Non-CALD parents of autistic children have previously described valuing proactive consultation about their wants and needs, believing ongoing consultation promoted effective parent-teacher partnerships (Galpin et al., 2018). Since our Chinese parents were deferential and undemanding towards teachers, parents may benefit from teachers themselves pre-emptively seeking parents’ views and expertise about their autistic children. Before schools consult with parents, it may be worthwhile considering whether current school communication channels (i.e., generic email addresses) make parents feel comfortable sharing confidential information about their children.

Self-education – especially awareness of rights in relation to school policies and procedures – has been suggested as integral for all parents of autistic children to prepare and encourage them to advocate (Boshoff et al., 2018; Lilley, 2019) – and Chinese parents are no exception here. Moreover, explicit teaching of other advocacy strategies, such as leadership and communication (Test et al., 2005), may be especially important for CALD parents who may not perceive themselves to be as self-efficacious as non-CALD parents (Galpin et al., 2018). It is important that the ‘hidden curriculum’, including ways in which parents can share their opinions with schools in an appropriate manner is important for the development of trusting and respectful partnerships. CALD families may also need additional time and other considerations to advocate effectively. Future research should focus on how we effectively support self-advocacy skills in CALD parents of autistic children.

Interpreters and translated materials should be available to parents. For equity, extended meeting times are recommended when using interpreters, as well as employing interpreters who have autism-specific knowledge (Sakai et al., 2019). Where possible, schools should also use simplified English and avoid acronyms. Finally, Australian teachers have reported low levels of cultural competency training and use of multicultural aides yet they reported that having access to professional development in this area would be useful (Syeda & Dresens, 2020). Autism-specific training, including teaching about the interplay of autism and culture, is recommended for *all teachers* to ensure educators have the skills to effectively educate all autistic children, in all settings, from all backgrounds. We must also further explore the best mechanisms for educating teachers about culture and autism to ensure knowledge and skills are translated into classrooms and schools.

### Limitations

This study has several limitations. First, our sample included a highly educated group of parents, many of whom spoke English, so the importance of language supports may have been understated. That said, most interviews were conducted in Cantonese/Mandarin suggesting that community languages were still preferred. Second, most children attended mainstream schooling so we may similarly have an underrepresentation of views from parents with autistic children in specialist settings. Finally, the concept of ‘losing face’ (Huang & Zhou, 2016; Liao et al., 2019) may have played a role in parents being reluctant to share personal information, especially related to their challenges, so we may have overlooked some latent themes here. However, because our interviews were all conducted by someone from the parents’ own cultural background, who has a strong presence in the Chinese autism community, the impact of this issue on our findings may have been reduced.

### Conclusion

These findings are important in showing how Chinese cultural values shape migrant Chinese parents’ expectations of education for their autistic children, and their interactions with teachers. We highlight how we might better foster parent-teacher partnerships for migrant Chinese parents and their autistic children. We hope this study contributes to the provision of targeted supports for these parents in order to strengthen partnerships with educators.
