# Correction: “They Were Saying That I Was a Typical Chinese Mum”: Chinese Parents’ Experiences of Parent–Teacher Partnerships for Their Autistic Children

**DOI:** 10.1007/s10803-022-05788-5

**Published:** 2022-10-20

**Authors:** Jodie Smith, Aspasia Stacey Rabba, Lin Cong, Poulomee Datta, Emma Dresens, Gabrielle Hall, Melanie Heyworth, Wenn Lawson, Patricia Lee, Rozanna Lilley, Najeeba Syeda, Emily Ma, Julia Wang, Rena Wang, Chong Tze Yeow, Elizabeth Pellicano

**Affiliations:** 1 Macquarie School of Education, Macquarie University, Sydney, NSW 2109 Australia; 2 Positive Partnerships, Forestville, Australia; 3 School of Allied Health, Human Services and Sport, La Trobe University, Melbourne, Australia; 4 Department of Psychology, Counselling and Therapy, School of Psychology and Public Health, La Trobe University, Melbourne, Australia; 5 Department of Clinical, Educational and Health Psychology, University College London, London, UK; 6 School of Educational Psychology and Counselling, Faculty of Education, Monash University, Melbourne, Australia

## Correction to: Journal of Autism and Developmental Disorders 10.1007/s10803-022-05748-z

Please note the following corrections to this article:

- The main title should have title case capitalisation with the first clause in speech marks. The correct title should read: “They Were Saying That I Was a Typical Chinese Mum”: Chinese Parents’ Experiences of Parent-Teacher Partnerships for Their Autistic Children.
- Reframing Autism should be an additional affiliation for co-author Melanie Heyworth.
- Co-author Chong Yeow should have a middle name (Chong Tze Yeow).
- The sentence “Ethical approval was gained from [BLINDED FOR REVIEW]” should be deleted from the Procedure section (page 3).
- The word ‘written’ should be deleted from the following sentences: “Once parents had provided written informed consent” (Procedure, page 3) and “Written informed consent was gained from all participants” (Declaration, page 11).
- The Theme 1 subheading (Results, page 6) should be in title case. The heading should read: “Theme 1: “Children are Your Heart”
- Under Theme 2 (Results, page 7), the following sentences should be amended:
  - “They expressed the view that “Australia teachers don’t want to talk about the bad things” should read: “They expressed the view that “Australian teachers don’t want to talk about the bad things”.
  - “They [teachers] just tell you that that the classroom is not for you” should read: “They [teachers] just tell you that the classroom is not for you”
- Under Theme 4 (Results, page 9), the following sentence should be amended:
  - “Because we are from Chinese culture” should read: “Because we are from a Chinese culture”.

